# Whole-body magnetic resonance imaging (WB-MRI) for cancer screening in asymptomatic subjects of the general population: review and recommendations

**DOI:** 10.1186/s40644-020-00315-0

**Published:** 2020-05-11

**Authors:** Fabio Zugni, Anwar Roshanali Padhani, Dow-Mu Koh, Paul Eugene Summers, Massimo Bellomi, Giuseppe Petralia

**Affiliations:** 1 Division of Radiology, IEO European Institute of Oncology IRCCS, Via Giuseppe Ripamonti 435, 20141 Milan, Italy; 2 Paul Strickland Scanner Centre, Mount Vernon Cancer Centre, Rickmansworth Rd, Northwood, HA6 2RN UK; 3 Department of Radiology, The Royal Marsden Hospital (Surrey), Downs Rd, Sutton, SM2 5PT UK; 4 Department of Oncology and Hemato-Oncology, University of Milan, Via S. Sofia, 9/1, 20122 Milan, Italy; 5 Precision Imaging and Research Unit, Department of Medical Imaging and Radiation Sciences, IEO European Institute of Oncology IRCCS, Via Giuseppe Ripamonti 435, 20141 Milan, Italy

**Keywords:** Whole-body imaging, Whole body screening, Magnetic resonance imaging, MRI, Incidental findings, Cancer screening

## Abstract

**Background:**

The number of studies describing the use of whole-body magnetic resonance imaging (WB-MRI) for screening of malignant tumours in asymptomatic subjects is increasing. Our aim is to review the methodologies used and the results of the published studies on per patient and per lesion analysis, and to provide recommendations on the use of WB-MRI for cancer screening.

**Main body:**

We identified 12 studies, encompassing 6214 WB-MRI examinations, which provided the rates of abnormal findings and findings suspicious for cancer in asymptomatic subjects, from the general population. Eleven of 12 studies provided imaging protocols that included T1- and T2-weighted sequences, while only five included diffusion weighted imaging (DWI) of the whole body. Different categorical systems were used for the classification and the management of abnormal findings.

Of 17,961 abnormal findings reported, 91% were benign, while 9% were oncologically relevant, requiring further investigations, and 0.5% of lesions were suspicious for cancer.

A per-subject analysis showed that just 5% of subjects had no abnormal findings, while 95% had abnormal findings. Findings requiring further investigation were reported in 30% of all subjects, though in only 1.8% cancer was suspected. The overall rate of histologically confirmed cancer was 1.1%.

**Conclusion:**

WB-MRI studies of cancer screening in the asymptomatic general population are too heterogeneous to draw impactful conclusions regarding efficacy. A 5-point lesion scale based on the oncological relevance of findings appears the most appropriate for risk-based management stratification. WB-MRI examinations should be reported by experienced oncological radiologists versed on WB-MRI reading abnormalities and on onward referral pathways.

## Background

**Fig. 1:**
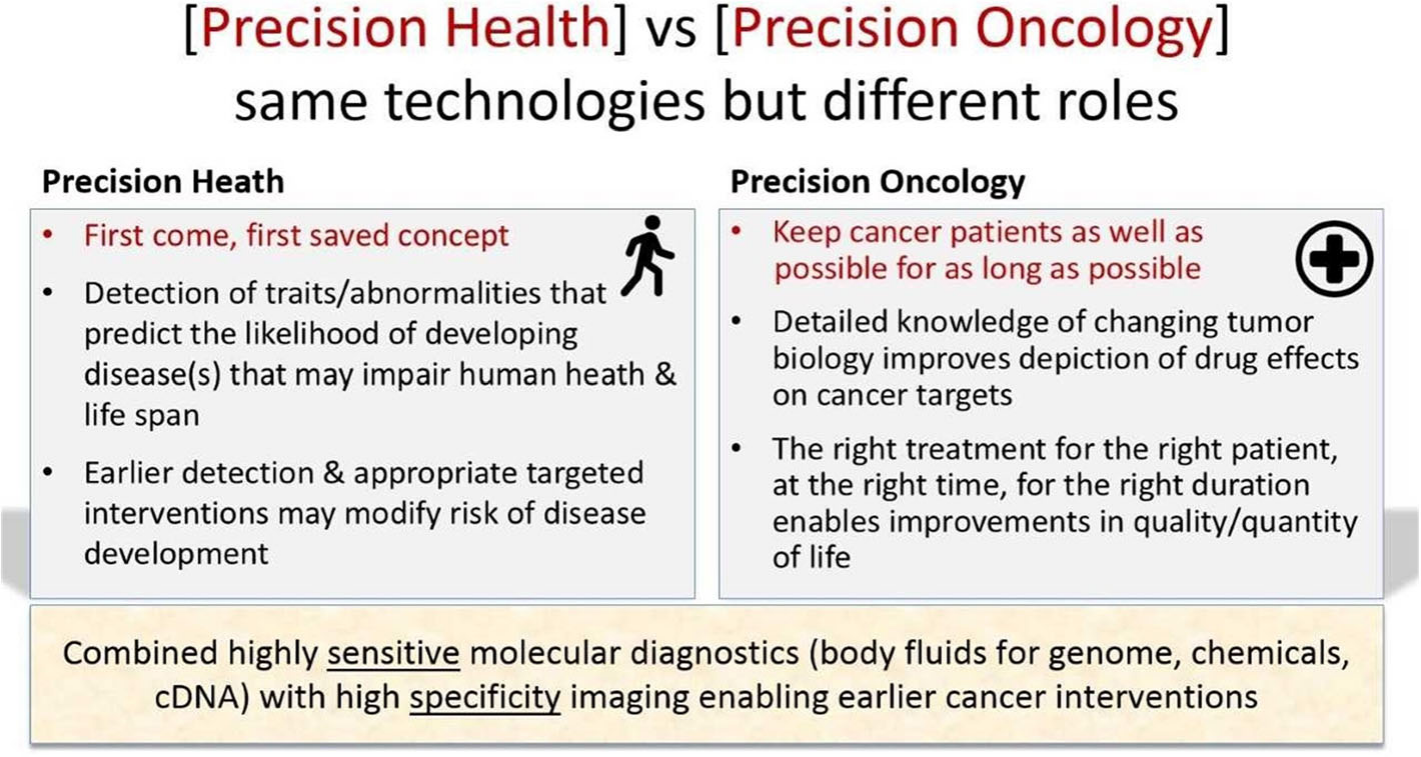
[Precision Health] vs [Precision Oncology] same technologies but different roles

Whole-body magnetic resonance imaging (WB-MRI) has become established for the management of patients with multiple epithelial and non-epithelial cancers, and recently its use has been extended to early cancer detection in subjects with cancer predisposition syndromes [1, 2]. However, there is increasing interest in applying WB-MRI to detect cancers in the general population given the high sensitivity of the method that is free from ionising radiation. The premise being that earlier detection and appropriate targeted interventions can modify the risk of disease development and so promote precision health. In this setting, imaging modalities can be combined with other molecular diagnostics, such as genomic profiling, biochemical tests and circulating cell-free DNA. Highly sensitive molecular diagnostics can be used to stratify each subjects’ risk of developing malignant cancer. Thereafter, highly specific imaging tests such as WB-MRI are used to detect and characterise abnormalities in these subjects, allowing both early diagnosis of malignant tumours for which interventions or surveillance is warranted. This use of WB-MRI here is distinct to its current role for promoting precision oncology (Fig. 1). In this review, we first summarise the roles of WB-MRI in oncology and cancer predisposition syndromes, before examining the feasibility of using this technique to more general population screening.

### Guideline recommendations and key uses in known cancers

The International Myeloma Working Group and the British society of Haematology recommend the use of WB-MRI for the detection and staging of multiple myeloma (Grade A recommendation, GR A) [3], as well as for the detection of relapsed disease prompted by rising serum paraprotein levels. Additionally, more regular use of WB-MRI is recommended for the follow-up of oligo-secretory/non-secretory disease and for patients with extramedullary disease (Level of Evidence 1B, LE 1B) [4]. Guidelines have been published for the use of WB-MRI in multiple myeloma (Myeloma Response Assessment and Diagnosis System, MY-RADS) [5], including standardized acquisition protocols, which rely on both morphological and diffusion weighted imaging (DWI) sequences..

In light of the good diagnostic performances for the detection of metastases in several articles [6–8] the German Dermatology Society, the Dermatologic Cooperative Oncology Group and the updated Swiss Guidelines suggested the use of WB-MRI as an alternative to 18-flurodeoxyglucose (FDG) PET/CT for the staging of high-risk and metastatic (stage III or IV) melanoma (LE 1A, GR B), and for the follow-up of stage IIC or higher melanoma patients (LE 4) [9, 10].

WB-MRI is also being increasingly used for the management of patients where there is a propensity for tumour spread to the bone marrow including prostate and breast cancers [1]. The European Association of Urology (EAU) recognized that WB-MRI is more sensitive than choline PET/CT and bone scan for detecting bone metastases in high-risk prostate cancer patients [11], but acknowledges the limited availability of the technique [12]. The Advanced Prostate Cancer Consensus Conference (APCCC) noted that WB-MRI, although less widely used, is more sensitive for detecting bone metastases than conventional techniques such as computed tomography and planar bone scans [13]. Recently, an ASCO consensus guideline outlined a number of clinical scenarios where next-generation imaging including PET/CT, PET/MRI, or WB-MRI could have management impacts in men with advanced prostate cancer [14]. Metastasis Reporting and Data System for Prostate Cancer (MET-RADS-P) [15] guidelines provided a standardization of acquisition protocols, based on morphological and DWI sequences, and a guidance for image interpretation and structured reporting.

The application of WB-MRI in breast cancer (BC) patients can be applied to two specific clinical subgroups [16]. The first comprises BC patients with bone-predominant or bone-only metastatic disease, where WB-MRI is able to show progressive disease earlier than computed tomography (CT) and bone scans, enabling treatment changes at lower burdens of progressing disease [17, 18]. The second comprises women who develop BC during pregnancy. As a radiation-free imaging technique requiring no contrast medium administration, WB-MRI has been proposed as the technique of choice for systemic staging of pregnant women developing BC [19, 20].

There is growing use of WB-MRI for the follow-up of lymphoma patients with non-avid or variable FDG PET/CT avidity where WB-MRI has superior diagnostic performance to FDG-PET/CT [21]. Furthermore, WB-MRI has a diagnostic performance comparable to FDG-PET/CT in FDG avid lymphoma patients [22]. The enthusiasm for using WB-MRI as a surveillance method in children and younger patients is motivated by the clinical need to minimise radiation exposure following the ALARA (As Low As Reasonably Achievable) principles of radioprotection [23].

Finally, two large multicentre prospective studies have been recently published, comparing the diagnostic accuracy and efficiency of WB-MRI-based staging pathways with standard pathways in colorectal and lung cancer [24, 25]. In both studies, WB-MRI staging pathways had similar accuracy to standard pathways and reduced staging time and costs.

### Guideline recommendations in Cancer predisposition syndromes

Several international guidelines recommend WB-MRI for the early cancer detection in individuals with cancer predisposition syndromes where regular surveillance is necessary. These recommendations are underpinned by the lack of ionizing radiation exposure using WB-MRI and the good diagnostic performance for disease detection, with a sensitivity ranging from 50 to 90%, and a specificity ranging from 93 to 95%, as described in the largest studies available [24–26].

In the setting of Li-Fraumeni syndrome (LFS), guidelines developed by the National Comprehensive Cancer Network (NCCN) and by the American Association for Cancer Research (AACR) indicate annual WB-MRI along with brain MRI with contrast (and breast MRI in women) as the techniques of choice for the surveillance of paediatric and adult subjects [27, 28]. Screening protocols that include WB-MRI for subjects with LFS have been also proposed by Australian and Canadian researchers [29, 30].

For children and adults with hereditary paraganglioma and pheochromocytoma syndromes, the AACR also recommends biennial screening using WB-MRI [31].

In patients with neurofibromatosis, WB-MRI showed good sensitivity in detecting the number, volume, and distribution of neurofibromata in a study of 247 subjects by Plotkin et al. [32]. In light of these results, the NCCN recently suggested the development of practical guidelines to introduce WB-MRI for the detection of malignant peripheral nerve sheath tumours and to establish a standardized, cost-efficient WB-MRI protocol for image acquisition [33].

In subjects with constitutional mismatch repair deficiency syndrome (CMMRD), a consensus statement by the Care for CMMRD Consortium and by the International Biallelic Mismatch Repair Deficiency Consortium recommends yearly WB-MRI from the age of six [34] to screen for development of cancers.

### Cancer screening in the general population

A meta-analysis [35] and systematic review [36] have recently summarized the diagnostic yields of WB-MRI in the population screening context, with particular focus on the prevalence of relevant and indeterminate findings. However, there are no evidence-based recommendations on the key issues such as imaging protocols and strategies for classifying and/or managing findings.

To address this short coming, we identified using PubMed searches and cross-checking of citations, 14 studies published between 2005 and 2020 describing the use of WB-MRI for cancer screening in asymptomatic subjects in the general population. For 12 of the 14 studies (6423 subjects) the intended purpose was or included cancer screening [37–48]. In the remaining two studies, the main purpose was the mapping of body fat (148 subjects) [49], or cardiovascular disease screening (138 subjects) [50], with any lesion suspicious for cancer described as incidental findings. These two studies were not considered for this review. We note that the 209 subjects included in the pilot study by Perkins et al. [39] were included also in the larger study by Hou et al. [40]. Therefore, this was considered in the overall count of screened subjects, as reported in Table 1.

**Table 1:** overview of the 12 studies reporting WB-MRI in asymptomatic subjects of the general population

			*WB-MRI protocol*								*Per-lesion analysis*								*Per-subject analysis*							*Cancer detection*			
Author	Year	Country	Magnet strenght	T1W TSE	T1W GE	T2W FS	T2W	DWI	Contrast	Cardiovascular sub-protocol	Classification method	All findings	Not relevant		≥ Relevant		Highly relevant		n° of WB-MRI examinations	Entirely normal (no findings)		Abnormal findings reported		With relevant findings (requiring further evaluation)		Suspected malignant cancers		Confirmed malignant cancers	
***Goehde***^***37***^	2005	GER	1.5		WB		S	S	Y	cardiac, WB-MRA	binary: 1 (non-relevant), 2 (relevant)	329	233	70,8%	96	29,2%			298					82	31.0%	1	0,3%	1	0.3%
***Baumgart***^***38***^	2007	GER	1.5		WB		S		Y	cardiac, WB-MRA	–								1007							4	0,4%	4	0.4%
***Lo***^***41***^	2008	HKG	3.0		WB	S	WB				binary: 1 (non-relevant), 2 (relevant)	234	210	89,7%	24	10,3%			132	8	6,1%	124	93,9%	24	18.2%	4	3,0%	4	3.0%
**Takahara**^**42**^	2008	NED	1.5		WB		WB	WB			–								10					1	10.0%	1	10,0%	1	10.0%
***Hegenscheid***^***43***^	2013	GER	1.5		WB	WB	S	S	optional	cardiac, WB-MRA	categorical system: 1(non-relevant), 2 (relevant benign), 3 (relevant unclear), 4 (relevant malignant)	13,455	12,403	92,2%	990	7,4%	62	0,5%	2500					787	31.5%	62	2,5%		
***Cieszanowski***^***44***^	2014	PLN	1.5		WB	WB					categorical system: 1 (non-relevant), 2 (moderately or potentially relevant), 3 (relevant)	3375	2997	88,8%	378	11,2%	15	0,4%	666	7	1,1%	659	98,9%			7	1,1%	7	1.1%
***Tarnoki***^***45***^	2015	GER	3.0	WB	WB	WB		WB	Y	WB-MRA	categorical system: 1 (non-relevant), 2 (requiring further evaluation), 3 (relevant)	68	44	64,7%	24	35,3%	1	1,5%	22	2	9,1%	20	90,9%	15	68.0%	1	4,5%		
***Ulus***^***46***^	2016	TUR	1.5			WB	WB	S	optional		–								116	33	28,4%	83	71,6%	12	10.3%	3	2,6%	2	1.7%
***Saya***^***47***^	2017	UK	1.5		WB	WB		WB			categorical system: 1 (definitely benign), 2 (likely to be benign), 3 (equivocal), 4 (likely to be malignant), 5 (definitely malignant)								44					8	18.2%	0	0,0%	0	0%
***Lee***^***48***^	2018	KOR	1.5		WB	WB					categorical system: 1 (benign), 2 (requiring further evaluation), 3 (malignant)	500	290	58,0%	210	42,0%	6	1,2%	229	16	7,0%	213	93,0%			6	2,6%	2	0.9%
Perkins*^39^	2018	USA	3.0		WB		WB	WB	N	cardiac	–								209*					70	33.5%	4	1,9%	4*	1.9%
**Hou**^**40**^	2020	USA	3.0		WB		WB	WB	N	cardiac	–								1190									20	1.7%
**Total**												17,961	16,336	**91,0%**	1625	**9,0%**	84	**0.5%**	6214	66 / 1165	**5,7%**	1099 / 1165	**94,3%**	**999 / 3331**	**30,0%**	93 / 5233 **1.8%**		41 / 3692 **1.1%**	

Abbreviations: T1W = T1 weighted; T2W = T2 weighted; TSE = turbo spin echo; GE = gradient echo; FS = fat saturated; DWI = diffusion weighted imaging; S = single anatomic segment; WB = whole-body; WB-MRA = whole-body magnetic resonance angiography

* subjects included in this study are also included in the study by Hou et al. Therefore, they were not counted in the overall number of WB-MRI examinations, nor in the overall number of confirmed malignant cancers

#### Imaging protocol

- *Literature review*

In all 12 studies for cancer screening, the anatomical coverage included head, neck, chest, abdomen and pelvis; however, the lower limbs were included in nine studies (Supplementary Fig. 1). For all 12 studies it was possible to obtain detailed information regarding the orientation of the acquired images and the types of sequences used in the WB-MRI protocol, which are summarized in supplementary Table 1 and supplementary Fig. 1. In nine [39–41, 43–45, 47, 48], both T1 and T2 weighted images were acquired across the whole body, while in the remaining three studies, just one morphological sequence was acquired (Table 1 and supplementary Table 1). Whole body DWI sequences were utilized in just five studies [39, 40, 42, 45, 47]. All studies provided detailed information regarding the WB-MRI protocol used for cancer screening. This illustration provides a synthesis of the anatomical coverage and the image orientation used for the standard unenhanced examination, in the different body regions [Additional Fig. 1]. Additional sub-protocols for the evaluation of specific organs were performed in six studies.

Whole-body T1-weighted images were acquired in 11 studies [37–45, 47, 48], always using Gradient Echo (GRE) sequences, while Turbo Spin-Echo (TSE) sequences were used only in one of them, in addition to GRE. Whole-body T2-weighted images were acquired in eight studies using TSE sequences: with fat-suppression via Inversion Recovery techniques in five, with both fat-suppressed and unsuppressed acquisitions in one, and without fat suppression in two. Whole body DWI was performed in five studies [39, 40, 42, 45, 47], always in addition to the morphological T1 and/or T2-weighted imaging.

Additional regional oncologic MRI sub-protocols were performed in six out of 11 studies (Supplementary Table 1), including comprehensive multi-sequence brain MRI in four studies [37, 39, 40, 43], MR colonography in two [37, 38], MRI mammography in one [43] and prostate MRI in two [39, 40] (Supplementary Fig. 1). Six studies made use of sub-protocols for the non-oncologic evaluation of the cardiovascular system [37–40, 43, 45]. Supplementary Table 1 provides further details regarding the protocols used in each study.

Intravenous contrast agent was administered in three studies where WB-MRI was performed for cancer screening. However, its use was motivated by additional sub-protocols requiring contrast administration performed in the same sitting, including cardiac MRI, MR angiography and MR colonography [37, 38, 45]. In a fourth study, contrast was administered in those patients who accepted to undergo optional cardiac MRI, whole-body MR angiography or MR mammography [43]. In one study, intravenous contrast agent was administered in a minority of subjects (12 out of 116) to further characterise suspicious findings detected by the unenhanced sequences [46].

b) *Evidence Synthesis and recommendations*

WB-MRI scanning protocols for cancer screening are the analogous to protocols laid out for metastasis detection in advanced prostate cancer (MET-RADS-P) [15] and multiple myeloma (MY-RADS) [5], with minor modifications. Morphologic imaging forms the basis of WB-MRI protocols in MET-RADS-P and MY-RADS guidelines, with GRE T1-weighted images in axial or coronal orientation considered mandatory from head to mid-thigh for MET-RADS and to the knee for MY-RADS, while axial TSE T2-weighted images without fat suppression are considered optional. For cancer screening protocols, both T1-weighted and T2-weighted images without fat suppression are required for the optimal localization and characterisation of findings. T1-weighted imaging can be performed using a GRE Dixon sequence, allowing fat-only, water-only and relative fat-fraction images to be derived [51]. While T2-weighted sequences with fat suppression have traditionally been used in musculoskeletal studies, T2-weighted sequences without fat suppression seem more suitable for oncological studies and more time efficient, as recommended by MET-RADS-P and MY-RADS guidelines, and are therefore suggested for WB-MRI cancer screening..

Inclusion of the lower limbs is mandatory in WB-MRI protocols for cancer screening in subjects with cancer predisposition syndromes, such as Li-Fraumeni syndrome [30], due to high incidence of soft tissue cancers. Since malignant lesions in the lower limbs have not been reported in any studies of WB-MRI for cancer screening in the general population, a protocol that covers from head to mid-thigh is sufficient for cancer screening.

While the use of gadolinium-based contrast agents can increase the diagnostic performance of WB-MRI in some body regions (particularly the brain), it also represents a more invasive approach to imaging with unclear benefits in asymptomatic subjects [52]. The largest study included in our review (2500 subjects) highlights the low diagnostic yield of contrast enhanced sub-protocols, with only three tumours diagnosed by MRI mammography and no tumours detected on post-contrast T1-weighted imaging performed for whole-body MRI angiography (WB-MRA) [43]. In fact, most authors have avoided the use of contrast agent in general cancer screening, except when cardiovascular risk is also being assessed or when abnormalities are seen during WB-MRI examinations requiring supplementary contrast enhancement to arrive at a diagnosis. The issues of gadolinium deposition in the brain and other body tissues [53], and the discomfort related to intravenous puncture, represent further disincentives for its use in general cancer screening, therefore the use of contrast agents is not recommended.

Diffusion sequences have shown high sensitivity for cancer detection across multiple body regions; however, only seven studies included in our review made use of this technique. Outside the brain, DWI sequences were limited to the upper abdomen in two studies and used for whole-body evaluation in five studies [39, 40, 42, 45, 47]. Notably, the studies including DWI were published after year 2009, whereas three out of five studies not using DWI were published before 2009. It is interesting to note that recognition of the usefulness of DWI for cancer imaging emerged from a consensus conference of the International Society for Magnetic Resonance in Medicine [54] published in 2009. Progress in MRI technology has both improved DWI image quality and reduced acquisition times, making this technique highly suitable for whole-body imaging. Therefore, DWI should be used, pending future studies investigating WB-MRI with DWI for general cancer screening.

With existing commercial MR hardware and sequences, the proposed mandatory components could be acquired in under thirty minutes (Table 2). Additional regional assessments with specific sequences, for example brain examinations with FLAIR sequences and lungs evaluation with short echo-time GRE. Additional T1 weighted, and T2 weighted images with fat suppression of the spine, are recommended for metastasis detection by MET-RADS-P and MY-RADS guidelines, but this may not be necessary in the setting of cancer screening; in fact, only four screening studies include sagittal imaging of the spine.

**Table 2:** proposed WB-MRI protocol for cancer screening in asymptomatic subjects of the general population

	Sequence description	Characteristics	Recommendation
1	Whole-body (head to mid-thigh) T1W GRE, Dixon technique	Axial or coronal (5 mm slice thickness)	Mandatory
2	Whole-body (head to mid-thigh) T2W, TSE without fat- suppression	Axial or coronal (5 mm slice thickness)	Mandatory
3	Whole-body (head to mid-thigh) DWI, STIR fat suppression, contiguous slicing, multiple stations • ADC calculations with mono-exponential data fitting • 3D-MIP reconstructions of highest b-value images*	Axial (5 mm slice thickness) 2 b-values: • b50–100 s/mm^2^ • b800–1000 s/mm^2^)	Mandatory
Additional regional assessments:			
4	• Brain: T2W FLAIR	Brain: Axial (5 mm slice thickness)	Optional
5	• Lung: T1 GRE short echo-time single breath hold	Lung: Axial (< 3 mm slice thickness)	Optional
6	• Whole spine T1W, TSE	Sagittal (4–5 mm slice thickness)	Optional
7	• Whole spine STIR (preferred) or fat suppressed T2W	Sagittal (4–5 mm slice thickness)	Optional

W = weighted; TSE = turbo spin echo; STIR = short tau inversion recovery; GRE = gradient echo; DWI = diffusion weighted imaging; ADC = apparent diffusion coefficient; MIP = maximum intensity projection; FLAIR = FLuid Attenuated Inversion Recovery

* Whole-body rotational 3D MIP images rotating along the cranio-caudal axis (≤3 degrees of rotation per frame), displayed using an inverted grey scale

To avoid errors and reduce the demands on radiographers, we strongly recommend the composing of contiguous imaging blocks for each sequence, as well as the automated calculation of derived images (e.g. water, fat and fat fraction from Dixon images, and reconstruction of maximum intensity projections of the high b-value DWI images), when possible.

#### Reading and reporting

In a study on the diagnostic performance of WB-MRI for cancer screening in subjects with LFS, Anupindi et al. proposed that the examinations must be reported by radiologists with experience in oncologic WB-MRI [55]. We suggest extending this recommendation to WB-MRI for cancer screening also, where it is extremely important that readers are experienced enough to avoid harms through unnecessary additional testing on the one hand, and to have detailed knowledge of common cancer guidelines and of best practice recommendations, to appropriately advise subjects with relevant findings. To date, the number of WB-MRI examinations a radiologist should report to gain enough expertise is not known, as no study has formally addressed this issue. However, it is likely that the required expertise can be most readily be reached by oncological radiologists, who routinely report WB-MRI examinations in cancer patients. Where this may not be possible or practical, Greer et al. have suggested that centres with a low volume of WB-MRI examinations could benefit from central review of such examinations by more experienced readers [56].

#### Strategies for the classification of WB-MRI findings

- *Literature review*

Seven studies reported the use of categorical systems for the classification of findings. Two studies made use of a binary classification distinguishing between non-relevant (benign and not requiring further evaluation) or relevant findings (requiring further imaging or diagnostic workup) [37, 41]. Three studies classified findings into three categories, as either non-relevant (benign, not significant), relevant (requiring further evaluation) or highly relevant (malignant, highly significant) [44, 45, 48]. One study classified findings into four categories (non-relevant, relevant benign, relevant unclear, relevant malignant) [43], while the remaining study used five categories (definitely benign, likely to be benign, equivocal, likely to be malignant, definitely malignant) [47]. Findings related to cardiovascular diseases were reported in a separate section for the six studies that included cardiac or angiographic imaging sub-protocols, but these are not relevant to the current discussion, which is focused on oncologic findings.

b) *Evidence synthesis and recommendations*

Strategies adopted for classification of findings differed widely, rendering systematic comparison between studies difficult. For example, the binary classifications adopted in two studies [37, 41] does not describe the number of subjects with a strong suspicion for tumour, therefore reducing the interpretability of the results. Similarly, in one study [44] where three categories were used, the rate of highly relevant findings (0.4%) also included non-neoplastic findings requiring immediate referral, implying that the rate of oncologically relevant findings was lower. This difference may not be clear to subjects willing to undergo the examination, creating erroneous expectations regarding the performance of WB-MRI for cancer screening in the general population.

The adoption of a standardized structured report akin to disease specific MET-RADS-P and MY-RADS templates adapted for screening applications will likely improve reporting repeatability, as well as provide greater reproducibility and comparability across studies. Such a reporting template has yet to emerge for general population screening. We believe that a classification system based on five categories should be adopted at a lesion level to indicate the likelihood of malignancy in cancer screening setting. Category 1 and 2 for normal and benign findings, and categories 3, 4 and 5 for findings with increasing oncological relevance (Table 3). Stratification of the oncological relevance of findings would allow the application of different strategies for investigations and patient management.

**Table 3:** proposed classification system for findings detected by WB-MRI

Category	Likelihood of cancer
1	Normal
2	Benign
3	Equivocal
4	Suspicious
5	Very suspicious

1–2 = no follow-up

3–4-5 = follow-up or further investigation triggered by WB-MRI

#### Strategies for the management of WB-MRI findings

- *Literature review*

The management of relevant findings was only described in five studies, representing less than half of the reviewed papers. In three, detailed descriptions of the management of relevant findings was reported: Lo et al. [41] made use of additional imaging evaluations for specific body regions (ultrasound for thyroid nodules, CT for lung nodules, pancreatic and retroperitoneal lesions, contrast enhanced MRI for liver, kidney and prostate lesions, plain radiograph for long bones focal lesions); Ulus et al. [46] performed dedicated contrast enhanced MRI studies in the same sitting of WB-MRI for the majority of suspicious findings and used CT for lung nodules; Goehde et al. [37] made use of region specific imaging modalities (CT scans for lung nodules, MRI for brain, liver, kidney and bone lesions, sonography for thyroid nodules) and direct histopathological verification for clearly malignant masses (kidney). In the remaining two studies [43, 47], further management was discussed by a multidisciplinary board, but provided no descriptions of additional examinations undertaken.

b) *Evidence synthesis and recommendations*

The adoption of a standardised management of relevant findings represents a critical gap for the general use of WB-MRI for cancer screening. Given the high sensitivity of the technique, successful adoption of WB-MRI depends on having the means and methods to manage the entire range of findings generated by a single WB-MRI examination. Management should follow established guidelines for incidental findings in the different body regions as far as possible, such as those for lung nodules [57], renal cysts [58], pancreatic cysts [59] and the Radiology White Papers for Managing Incidental Findings on Abdominal and Pelvic CT and MRI [60], also requiring the establishment of specific onward referral pathways for all findings observed.

#### Abnormal findings in WB-MRI: per-finding and per-subject analysis

A per-finding analysis of the outcome of WB-MRI was possible in six studies (Table 1), which reported a total of 17,961 findings. From a per-finding perspective, 91% of reported findings were non-relevant and 9% were oncologically relevant (i.e. requiring further investigation). In the four studies that also provided the rate of highly relevant findings (i.e. suspicious for malignancy), this proportion reached 0.5% of all findings. The number of findings suspicious for malignancy reported in each study across the different body regions are summarized in Table 4. Notably, no suspicious tumours were reported in the lower limbs in the general population, despite coverage across 4800 examinations.

**Table 4:** Suspicious malignant cancers detected by WB-MRI in the published studies

Author	Head			Neck			Chest			Abdomen			Pelvis			Lower limbs			Spine			MRI mammography		
***Goehde***^***37***^	1	NC	brain suspicious lesion				1	NC	lung suspicious lesion	1	H	renal cell carcinoma												
***Baumgart***^***38***^							2	H	bronchial adenocarcinoma	5	H	renal cell carcinomas												
***Lo***^***41***^				1	H	thyroid follicular carcinoma	1	H	bronchial adenocarcinoma	1	H	renal cell carcinoma												
										1	H	neuronedocrine tumor												
**Takahara**^**42**^							1	H	lung cancer															
***Hegenscheid***^***43***^	1	NC	malignant head lesion	3	NC	malignant neck lesions				21	NC	malignant urinary tract lesions	17	NC	female genital malignant lesions				2	NA	malignant vertebral lesions	3	NA	malignant lesions
										7	NC	malignant abdominal organs lesions							8	NA	metastases (site not specified)			
***Cieszanowski***^***44***^	1	H	glioma				1	H	bronchial adenocarcinoma	2	H	renal cell carcinomas	1	H	ovarian tumor									
							1	H	metastases				1	H	testicular tumor									
***Tarnoki***^***45***^													1	NC	pararectal suspicious lesion									
***Ulus***^***46***^										1	H	renal cell carcinoma												
										1	H	adrenal carcinoma												
										1	H	pancreatic cystadenoma												
***Saya***^***47***^																								
***Lee***^***48***^				1	I	Tongue cancer				3	NC	suspicious renal lesions												
										1	I	renal cell carcinoma												
**Hou**^**40**^	1	I	optic nerve glioma	2	H	papillary thyroid carcinoma	2	H	lymphoma	5	H	renal cell carcinomas	6	H	prostate cancers									
							1	H	thymoma	1	H	low-grade intraductal papillary mucinous neoplasm	2	H	urinary bladder carcinomas									
**Total**	**4**			**6**			**10**			**51**			**28**			**0**			**10**			**3**		

H = histologically confirmed, I = confirmed by further imaging, NC = non confirmed, NA = not investigated

A per-subject analysis of the outcome of the WB-MRI was possible in five studies (Table 1). From a per-subject perspective, 94% of the WB-MRI examinations were reported to show some abnormal findings while 6% were entirely normal. Nearly 30% of all WB-MRI yielded oncologically relevant findings, while highly relevant findings arose in only 1.8% of people. Despite the high number of findings detected by WB-MRI, the rate of examinations that potentially lead to further diagnostic evaluations, such as further imaging studies, remains relatively low, around 30%. This highlights the ability of WB-MRI not only for lesion detection but also for the characterization of potential abnormalities.

#### Cancer detection

- *Literature review*

On a per-subject basis, across eleven studies [37–39, 41–48], a total of 93 WB-MRI examinations out of 5233 were reported as positive for malignancy (1.8%). Notably however, in the 10 studies [37–42, 44, 46–48] that reported the number of confirmed malignant cancers, these were ultimately established in 41 out of 3692 examinations (1.1%) (Table 1).

- *Evidence synthesis*

The cancer detection rate of WB-MRI in the general population is comparable to those observed in other cancer screenings. In a meta-analysis by Blanks et al. [61] showed a detection rate of 7.59 per 1000 subjects (0.8%) for breast cancer at prevalent screening with digital mammography. Notably, a meta-analysis by Ballinger et al. [62] conducted in subjects with Li-Fraumeni Syndrome undergoing surveillance with WB-MRI reported a much higher cancer detection rate of 7%. Therefore, WB-MRI for screening in the general population should be assessed keeping in mind that the likely low prevalence of malignant tumours in these subjects will influence the negative predictive value (NPV) of the examination. On the other hand, the presence of risk factors and relevant family history for cancer should be carefully collected, to allow personalised stratification of the subjects’ cancer risk.

By the same measure, before WB-MRI examination, subjects from the general population should be informed about both the low pre-test probability of detecting malignant cancer and the high likelihood of findings requiring follow-up investigations. The NPV for the presence of a malignant tumour will depend upon the sensitivity of WB-MRI and from the prevalence of such disease in the population being evaluated. A meta-analysis by Li et al., including 1067 patients with different tumour types from 13 studies, calculated a pooled per-patient sensitivity and specificity for the detection of primary and/or metastatic lesions by WB-MRI with DWI of 90 and 95%, respectively [26]: from these results we calculated a NPV of 96%. Considering the lower prevalence of malignant tumours (reported average < 2%) in asymptomatic subjects of the general population undergoing WB-MRI for cancer screening, as a consequence we would expect even higher NPV values for WB-MRI in cancer screening, also emphasising the need to adjust the threshold for prompting further investigations of incidental findings. Therefore, given the low probability of diagnosing malignant cancer, a high threshold should be applied when requiring additional diagnostic tests for abnormal findings in the general population, to avoid over-investigations. In-depth investigations should be considered only for definite abnormalities, for which onward diagnostic pathways should be planned according to existing guidelines and good practises.

#### Patient acceptability

Given the high frequency of “abnormal” findings at WB-MRI screening, importance should be given to the possible repercussions on quality of life and patient anxiety. In 2013, Schmidt et al. published the results of a survey conducted on 471 subjects from the SHIP study, who had been notified of the presence of potentially relevant findings [63]. Among these subjects, 10% reported strong distress while awaiting for WB-MRI results (six weeks) and 29% reported moderate to severe distress after receiving the results. The same authors examined the long-term impact on quality of life and depressive symptoms [64] by surveying 2188 subjects 2.5 years after WB-MRI and 2232 individuals who had not undergone WB-MRI. The survey did not detect significant differences in quality of life and depressive symptoms between the two groups, or between the subjects who had been notified with potentially relevant findings and those who had not. The authors concluded that, while WB-MRI can generate distress and anxiety in the short term, it is generally well accepted in the long term, with quality of life and subjective stress-levels comparable to those of other already existing cancer screening programs.

## Conclusions

Despite the heterogeneous methodology and the variable results of WB-MRI studies performed for cancer screening in the general population, we can make a few generalised conclusions:

- The typical imaging protocol comprises T1-weighted GRE, T2-weighted FSE (fast spin-echo) and diffusion weighted sequences, extending from the head to mid-thigh, with optional additional regional assessments. The administration of intravenous contrast agent is not recommended.
- Abnormal findings are expected in about 95% of screened subjects, about 30% of subjects would require further investigations but less than 2% would be reported as suspicious for malignant cancers.
- Findings should be classified using a categorical system, based on their likelihood of malignancy. It is important to set high thresholds for further investigations to minimize harms from diagnostic testing.
- Subject counselling on the high likelihood of incidental findings and the low likelihood of cancer detection together with established onward referral pathways are needed.
- Training is needed for reporting WB-MRI examinations; however, the number of examinations a radiologist should report to acquire this expertise still has to be investigated.

Guidelines are needed to establish common strategies for the classification and management of abnormal findings in studies using WB-MRI for cancer screening. The current experience is still too heterogeneous to draw meaningful conclusions regarding general efficacy. Future multisite studies should aim to provide the evidence that may pave the way to guidelines and recommendations for asymptomatic population screening.

## Supplementary information

**Additional File 1: Table 1.** summary of MR technology and protocols used for WB-MRI. This table provides a detailed overview of the types of sequences used by the 12 studies included in this review. For each body region, the different types of sequence performed are annotated, with reference to the anatomical orientation of the planes. Additional sub protocols are also described.

**Additional file 2: Figure 1.** Summary of body regions and imaging planes covered by the WB-MRI core protocols of the 12 studies included in this review. Additional dedicated sub-protocols performed for the evaluation of specific organs are shown on the right. Sub protocols requiring administration of contrast agents are marked by an asterisk.

## Data Availability

The datasets during and/or analysed during the current study available from the corresponding author on reasonable request.

## Abbreviations

WB-MRI: Whole-body magnetic resonance imaging
GR: Grade of recommendation
LE: Level of evidence
PET/CT: Positron emission tomography / computed tomography
CT: Computed tomography
LFS: Li Fraumeni syndrome
CMMRD: Constitutional mismatch repair deficiency
GRE: Gradient echo
TSE: Turbo spin echo
DWI: Diffusion weighted imaging
FLAIR: Fluid attenuated inversion recovery
NPV: Negative predictive value
