# Supplementary material for: Whole-body magnetic resonance imaging (WB-MRI) for cancer screening in asymptomatic subjects of the general population: review and recommendations
Source: Cancer Imaging. 2020 May 11;20:34. doi: 10.1186/s40644-020-00315-0 (PMC7216394; doi:10.1186/s40644-020-00315-0)
Supplement: Supplementary file 1 — Additional File 1: Table 1. summary of MR technology and protocols used for WB-MRI. This table provides a detailed overview of the types of sequences used by the 12 studies included in this review. For each body region, the different types of sequence performed are annotated, with reference to the anatomical orientation of the planes. Additional sub protocols are also described. [file 40644_2020_315_MOESM1_ESM.docx]

**Additional Table 1:** summary of MR technology and protocols used for WB-MRI

| **Authors** | **Magnet strenght (Tesla)** | **Average scanning time (minutes)** | **Contrast.** | **Core WB-MRI protocol** | | | | | | | | | | | | | | | | | | | | | | | | | | | | | | | | | | | | | | | | | | | | |  | **Additional sub-protocols** | | | | | |
| --- | --- | --- | --- | --- | --- | --- | --- | --- | --- | --- | --- | --- | --- | --- | --- | --- | --- | --- | --- | --- | --- | --- | --- | --- | --- | --- | --- | --- | --- | --- | --- | --- | --- | --- | --- | --- | --- | --- | --- | --- | --- | --- | --- | --- | --- | --- | --- | --- | --- | --- | --- | --- | --- | --- | --- |
|  |  |  |  | **Head** | | | | | | | **Neck** | | | | | | | **Chest** | | | | | | | **Abdomen** | | | | | | | **Pelvis** | | | | | | | | **Lower limbs** | | | | | | | **Spine** | |  | **Brain** | **Breast*** | **Colon*** | **Prostate** | **Heart*** | **Arteries** |
|  |  |  |  | **T1W** | | | **T2W** | | | **DWI** | **T1W** | | | **T2W** | | | **DWI** | **T1W** | | | **T2W** | | | **DWI** | **T1W** | | | **T2W** | | | **DWI** | **T1W** | | | **T2W** | | | **DWI** | **PD** | **T1W** | | | **T2W** | | | **DWI** | **T1W** | **T2W** |  | Comprehensive brain MRI | MRI mammography | MRI colonography | *Bi-parametric MRI* | Cardiac MRI | MRA* |
|  |  |  |  | *ax* | | *cor* | *ax* | | *cor* | *ax* | *ax* | | *cor* | *ax* | | *cor* | *ax* | *ax* | | *cor* | *ax* | | *cor* | *ax* | *ax* | | *cor* | *ax* | | *cor* | *ax* | *ax* | | *cor* | *ax* | | *cor* | *ax* | *ax* | *ax* | | *cor* | *ax* | | *cor* | *ax* | *sag* | *sag* |  | *multi-plane* | *ax* | *cor* | *Multi-plane* | *multi-plane* | *cor* |
| ***Goehde^37^*** | 1.5 | 50 | Y | X | | X | X | |  | X |  | | X |  | |  |  |  | | X | X | |  |  |  | | X |  | |  |  |  | | X |  | |  |  |  |  | | X |  | |  |  |  |  |  | X |  | X |  | X | X |
| ***Baumgart ^38^*** | 1.5 | 60 | Y | X | | X | X | |  |  |  | | X |  | |  |  | X | | X | X | |  |  |  | | X |  | |  |  |  | | X | X | |  |  |  |  | | X |  | |  |  |  |  |  |  |  | X |  | X | X |
| ***Lo ^41^*** | 3.0 | 33 | N | X | | X | X | |  |  |  | | X | X | |  |  | X | | X | X | |  |  | X | | X | X | |  |  | X | | X | X | |  |  |  |  | |  |  | |  |  |  | X |  |  |  |  |  |  |  |
| **Takahara ^42^** | 1.5 | 38 | N |  | | X |  | | X | X |  | | X |  | | X | X |  | | X |  | | X | X |  | | X |  | | X | X |  | | X |  | | X | X |  |  | |  |  | |  |  |  |  |  |  |  |  |  |  |  |
| ***Hegenscheid ^43^*** | 1.5 | ~45 | Optional | X | | X | X | | X | X | X | | X |  | | X |  | X | | X | X | | X |  | X | | X | X | | X | X |  | | X |  | | X |  | X |  | | X |  | | X |  | X | X |  | X | X |  |  | X | X |
| ***Cieszanowski ^44^*** | 1.5 | 50 | N |  | |  | X | | X |  |  | |  | X | | X |  | X | |  | X | | X |  | X | |  | X | | X |  | X | |  | X | | X |  |  |  | |  |  | | X |  |  | X |  |  |  |  |  |  |  |
| ***Tarnoki ^45^*** | 3.0 |  | Y |  | | X |  | | X | X |  | | X |  | | X | X |  | | X |  | | X | X |  | | X |  | | X | X |  | | X |  | | X | X |  |  | | X |  | | X |  |  |  |  |  |  |  |  |  | X |
| ***Ulus ^46^*** | 1.5 | 30 | Optional |  | |  | X | | X |  |  | |  | X | | X |  |  | |  | X | | X |  | X | |  | X | | X | X |  | |  | X | | X |  |  |  | |  |  | | X |  |  |  |  |  |  |  |  |  |  |
| ***Saya ^47^*** | 1.5 | 60 | N | X | | X | X | |  | X | X | | X | X | |  | X | X | | X | X | |  | X | X | | X | X | |  | X | X | | X | X | |  | X |  | X | | X | X | |  | X |  |  |  |  |  |  |  |  |  |
| ***Lee ^48^*** | 1.5 | 30 | N |  | | X |  | | X |  |  | | X |  | | X |  |  | | X |  | | X |  |  | | X |  | | X |  |  | | X |  | | X |  |  |  | | X |  | | X |  |  | X |  |  |  |  |  |  |  |
| **Perkins ^39^** | 3.0 |  | N | X |  | | X |  | | X | X |  | | X |  | | X | X |  | | X |  | | X | X |  | | X | X | | X | X |  | | X |  | | X |  |  |  | |  |  | |  |  |  |  | X |  |  | X | X |  |
| **Hou ^40^** | 3.0 |  | N | X |  | | X |  | | X | X |  | | X |  | | X | X |  | | X |  | | X | X |  | | X | X | | X | X |  | | X |  | | X |  |  |  | |  |  | |  |  |  |  | X |  |  | X | X |  |
| Note: T1W = T1 weighted, T2W= T2 weighted, DWI = Diffusion Weighted Imaging, PD = Proton Density, MRI = Magnetic Resonance Imaging, MRA = Magnetic Resonance Angiography, ax = axial, cor = coronal, sag = sagittal.  Sub-protocols requiring administration of contrast agent are indicated by an asterisk. | | | | | | | | | | | | | | | | | | | | | | | | | | | | | | | | | | | | | | | | | | | | | | | | |  |  |  |  |  |  |  |
