# Supplementary figures and images for: Whole-body magnetic resonance imaging (WB-MRI) for cancer screening in asymptomatic subjects of the general population: review and recommendations
Source: Cancer Imaging. 2020 May 11;20:34. doi: 10.1186/s40644-020-00315-0 (PMC7216394; doi:10.1186/s40644-020-00315-0)

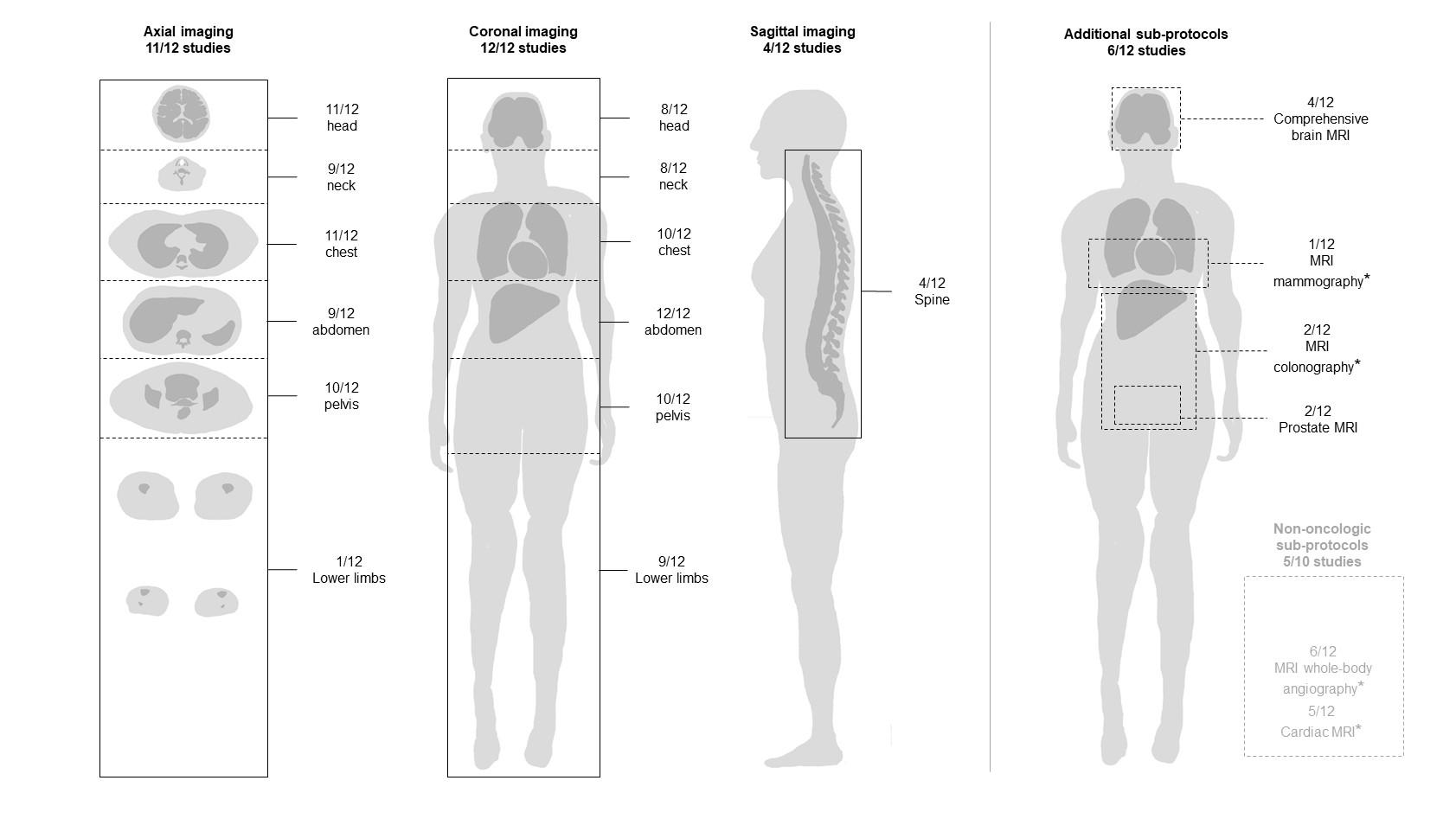

Supplement: Supplementary file 2 — Additional file 2: Figure 1. Summary of body regions and imaging planes covered by the WB-MRI core protocols of the 12 studies included in this review. Additional dedicated sub-protocols performed for the evaluation of specific organs are shown on the right. Sub protocols requiring administration of contrast agents are marked by an asterisk. [file 40644_2020_315_MOESM2_ESM.jpg]
